# Factors Associated with Quality of Life of Individuals Living with Parkinson’s Disease

**DOI:** 10.1093/geroni/igaf122.2475

**Published:** 2025-12-31

**Authors:** Odunayo Arogundade, Chelsea Spencer

**Affiliations:** Kansas State University, Manhattan, Kansas, United States; Kansas State University, Manhattan, Kansas, United States

**Keywords:** Parkinson’s Disease, quality of life, personal control, illness intrusion, spiritual well-being, social support

## Abstract

In the United States, Parkinson’s disease (PD) is the second most common neurodegenerative disorder, and PD is related to a decreased quality of life (QoL), increased health care utilization, and economic costs. This study aimed to establish the key psychosocial factors influencing QoL in individuals with PD to help develop evidence-based interventions. A cross-sectional study involving eighty-five (85) individuals living with PD was conducted. Data were collected using validated scales, including the Parkinson’s Disease Questionnaire (PDQ), Spiritual Quality of Life (SQL) scale, Personal Control (PC) scale, Illness Intrusion Rating (IIR) scale, and Quality of Life (QoL) scale. Descriptive and inferential statistics were used to analyze the data. The regression analysis showed that all the variables (personal control, illness intrusion, spiritual well-being, demographic characteristics, and social support) jointly accounted for 58.8% variance in QoL outcomes of people with PD. Higher PDQ and IIR scores were negatively correlated with QoL, while higher SQL and PC scores were positively associated with QoL. Gender and age variations also played a role in QoL outcomes. Interventions to help improve the QoL of individuals diagnosed with PD should take psychosocial factors, such as personal control, spiritual quality of life, as well as the progression of PD.

